# Entering the second decade: *FEBS Open Bio* in 2022

**DOI:** 10.1002/2211-5463.13343

**Published:** 2021-12-20

**Authors:** Duncan E. Wright, Miguel A. De la Rosa

**Affiliations:** ^1^ FEBS Open Bio Editorial Office Cambridge UK; ^2^ Institute for Chemical Research (IIQ) Scientific Research Centre Isla de la Cartuja (cicCartuja) Universidad de Sevilla‐CSIC Sevilla Spain

## Abstract

*FEBS Open Bio* continues to go from strength to strength, with 2021 perhaps marking its most exciting year. In this Editorial, the Editor‐in‐Chief Miguel A. De la Rosa looks back at all the new developments of 2021 and forecasts the outlook for 2022.

## Graphical abstract

In this Editorial, the Editor‐in‐Chief Miguel A. De la Rosa looks back at the exciting developments of *FEBS Open Bio* in 2021 and forecasts the outlook for 2022.

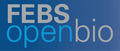

Last year was perhaps the most eventful since the journal’s beginning. In the last twelve months, we have started the following new initiatives:

‐ We published the first two of our ‘In the Limelight’ special sections, focussed on bioplastics and membraneless organelles, respectively;
‐ We started a new series called ‘Insights’, which summarizes research findings for a wider audience;
‐ We began publishing a series of interviews with members of our Editorial Board, under the moniker ‘An open chat with…’;
‐ We awarded two speed talk prizes for the first time at the FEBS Congress;
‐ We awarded the inaugural *FEBS Open Bio* Article Prize to Arpit Katiyar, the first author of the prize‐winning article [1].

We concluded 2021 with a special issue to celebrate the journal’s 10th anniversary issue, which included several editorials, and research and review articles especially commissioned to mark the occasion. For full details on the speed talk prizes and the 2021 *FEBS Open Bio* Article Prize, please refer to the editorial published in the 10th anniversary issue [2]. In this editorial, we take a closer look at this exceptional year in the journal’s history and share the plans for 2022.

## Welcome to the new members of the Editorial and Editorial Advisory Board

In 2021, we welcomed So Nakagawa (Junior Associate Professor at the Department of Molecular Life Science, Tokai University School of Medicine, Tokyo, Japan) and Ingela Lanekoff (Associate Professor in the Department of Chemistry – BMC, Uppsala University, Uppsala, Sweden) to the Editorial Board.

**Figure:**
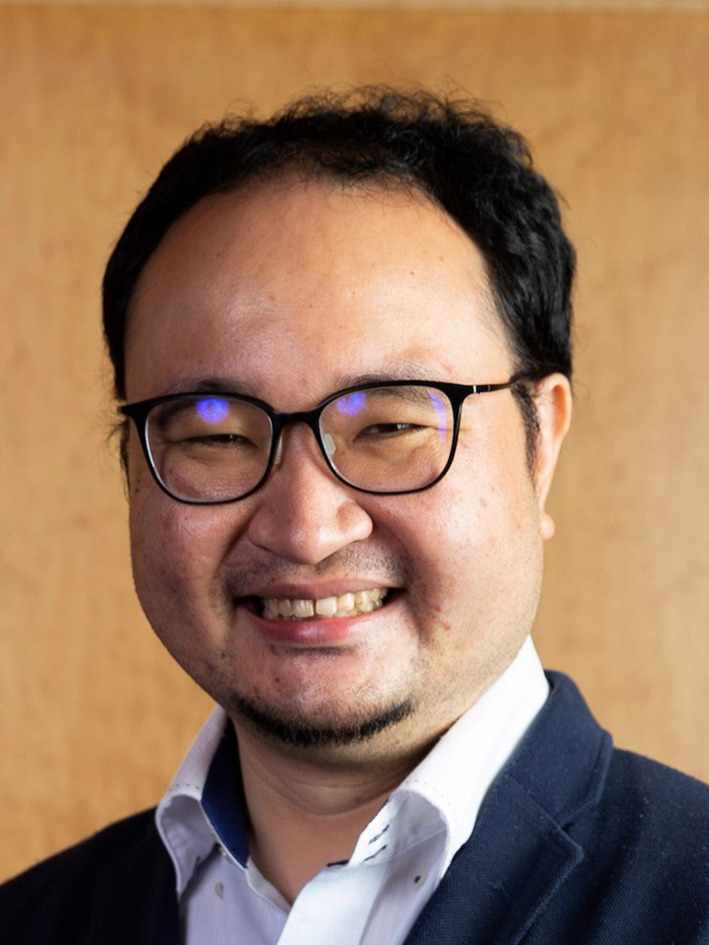
So Nakagawa

Prof. So Nakagawa obtained his Ph.D. from Tokyo Medical and Dental University in 2008. He completed postdoctoral training at National Institute of Genetics and Harvard University. He moved to Tokai University School of Medicine as Assistant Professor in 2013 and was promoted to Junior Associate Professor in 2018.

He has been working on comparative and evolutionary genomic studies, including translation initiation mechanisms, multigene families and retrotransposons. In particular, he is recently interested in virus and host co‐evolution. He conducts evolutionary studies of various retroviruses, including endogenous retroviruses (ERVs) and de novo genes in host species derived from ERVs. He also works on molecular evolution of coronaviruses including SARS‐CoV‐2 and filoviruses, including Ebola viruses.

**Figure:**
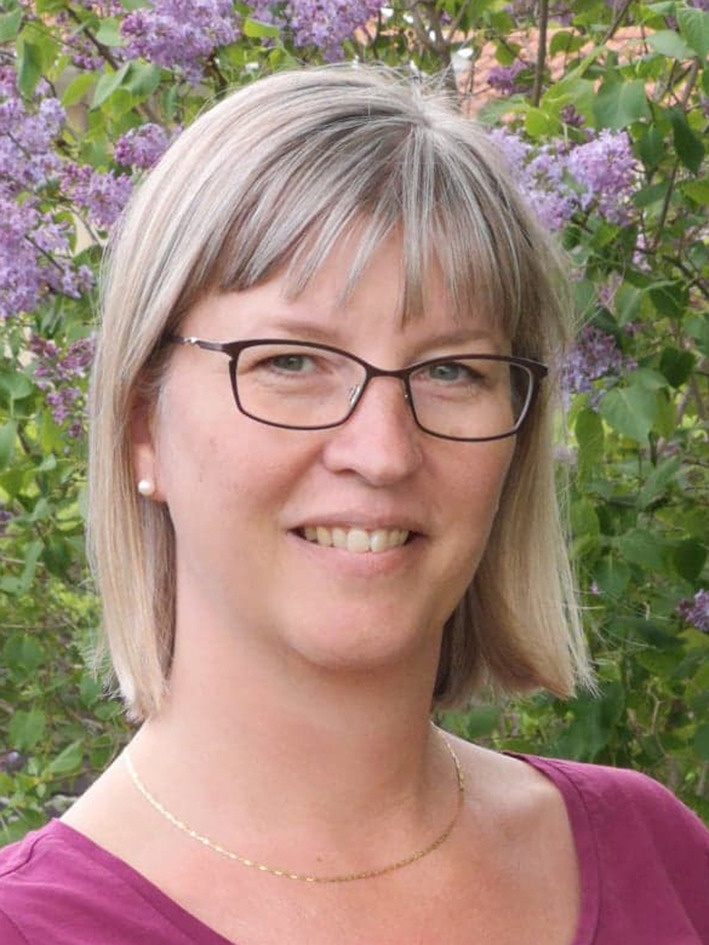
Ingela Lanekoff

Dr. Ingela Lanekoff completed her Ph.D. in 2011 at the University of Gothenburg in Sweden and spent two years as a postdoctoral research associate at Pacific Northwest National Laboratory in the United States. In 2014, she started her own research group at Uppsala University in Sweden, where she was recently appointed full Professor of Analytical Chemistry. The Lanekoff research group works on developments and applications of analytical approaches for spatially resolved and in‐depth analysis of small molecules, such as metabolites and lipids. In particular, they perform quantitative mass spectrometry imaging using nanospray desorption electrospray ionization and in‐depth spatial metabolomics with surface sampling capillary electrophoresis on thin tissue sections and cells to realize metabolites involved in health and disease.

In 2021, we also appointed 10 new members to the journal’s Editorial Advisory Board (EAB), bringing the total to 37. The EAB is instrumental in helping us ensure timely and thorough peer review for submissions to the journal, and we greatly appreciate their hard work and commitment. We warmly welcome the following to the EAB:

- Aditi BhargavaUniversity of California San Francisco, San Francisco, CA, USA
- Lukas CajanekMasaryk University, Brno, Czech Republic
- Jobichen ChackoNational University of Singapore, Singapore
- Sherien M. El‐DalyNational Research Centre, Cairo, Egypt
- Kazumi HiranoNational Institute of Advanced Industrial Science and Technology (AIST), Japan
- Gaetano LetoUniversity of Palermo, Italy
- Mozart MarinsUniversity of Ribeirão Preto, Brazil
- João Ramalho‐SantosUniversity of Coimbra, Portugal
- Christine SchwartzUniversity of Wisconsin‐La Crosse, La Crosse, WI, USA
- Zhen SunMemorial Sloan Kettering Cancer Center, USA

## New developments in 2021

### In the Limelight and the 10^th^ anniversary

In April 2021, we published the journal’s first ‘In the Limelight’ issue, which focussed on bioplastics and accompanied the FEBS Special Session on Science & Society – Plastics: revolution, pollution and substitution at the 45^th^ FEBS Congress. In this issue, Raffaele Porta, OF Department of Chemical Sciences, University of Naples ‘Federico II’, Italy, provided a comprehensive overview of the plastics revolution that began in the 1940s and resulted in the long‐term problem of global plastics pollution, before introducing the potential of biodegradable and/or bio‐based plastics as a solution to this problem [3]. Oliver Bajt, of Marine Biology Station, National Institute of Biology, Piran, Slovenia, presented a disconcerting summary of the detrimental effects of microplastics on marine life [4], emphasizing the urgent need for change. Paola Fabbri and colleagues at Alma Mater Studiorum Università di Bologna, Italy, contributed an enlightening article on the production of various bioplastics and the future of the bioplastics industry [5]. Finally, Frédéric Debeaufort of the University of Burgundy, Dijon Cedex, France, describes how certain waste products from the food industry may be used to produce sustainable food packaging, highlighting how biotechnology can be used to provide innovative solutions to the challenges we face today [6].

Our second ‘In the Limelight’ section was published in September 2021 and focussed on membraneless organelles. In the first article, Douglas V. Laurents of Instituto de Química Física Rocasolano examines the evidence for whether polyproline II (PPII) helices modulate biomolecular condensates [7]. In the second article, Irantzu Pallarès, Salvador Ventura, and colleagues at Universitat Autònoma de Barcelona, Spain, provide a comprehensive overview of the identification and function of prion‐like proteins, which, in addition to being implicated in certain diseases, are also associated with the formation of functional membraneless protein–nucleic acid coacervates [8]. Finally, Irene Díaz‐Moreno and colleagues at the University of Seville discuss the possible role of cytochrome c in nuclear condensate formation and nucleosome assembly [9].

Our third special issue of the year was published to commemorate the journal’s 10^th^ anniversary [2]. We were delighted at the number of editors and returning authors who agreed to contribute review articles or their latest research for consideration of inclusion, and the final line‐up is a truly exciting mix of articles on diverse and highly relevant topics. In addition, the issue includes editorials on the stories behind the journal’s genesis and development. Please refer to our introductory editorial in last month’s issue for full details on the contents and conception of the 10^th^ anniversary issue [2].

### Editor profiles and Insights

In 2021, we began publishing a series of interviews with members of our prestigious Editorial Board (dubbed ‘An open chat with…’). This series is coordinated by Beáta Vertessy, who gave the inaugural interview, published in the February 2021 issue [10]. In this interview, Beata discusses how her academic start in chemical engineering has been beneficial for her subsequent research in the uracil‐DNA field, her group’s discovery of the significance of uracil‐DNA in fruit fly development and how next‐generation genome‐wide sequencing technologies have actually impeded our understanding of DNA chemistry [10].

For our second fascinating interview, published in October this year, we spoke to Stuart Ferguson on his research on cytochrome assembly, the perils of online teaching and the possible effects of Brexit on Horizon Europe and scientific research in the UK generally [11]. We are extremely grateful to our editors for sharing their thoughts with us and hope that this series will be of interest to a wide readership. In 2022, we look forward to publishing interviews with Takashi Gojobori and Laszlo Nagy, among others.

To present articles published in the journal to a lay audience, we started a new series of articles called ‘Insights’ coordinated by our editor Cornelia de Moor. To date, we have published four Insight articles, which cover subjects as diverse as the role of the GAD67 protein in fear in rats [12, 13], the use of CRISPR to generate mutations in multiple copies of a gene [14, 15], the use of miRNAs as biomarkers for gastric cancer [16, 17] and the association between extracellular vesicles and susceptibility to social stress in mice [18, 19].

### Journal performance

The primary role of the journal is the assessment and publication of original research articles, and in 2021, we published 224 original research articles (including Methods articles), many of which have already been well cited and downloaded. We are pleased that the visibility of articles published in *FEBS Open Bio* continues to increase, with total downloads increasing dramatically year on year: by the end of October, downloads in 2021 were already 37% greater than in the whole of 2020. As a signatory of the San Francisco Declaration on Research Assessment, we strive to promote article‐level metrics, rather than those focussed on the journal. One of the most downloaded and cited articles published in *FEBS Open Bio* in 2021 is the excellent article ‘Seeded assembly *in vitro* does not replicate the structures of α‐synuclein filaments from multiple system atrophy’, authored by Sjors H. W. Scheres and colleagues [20]. We feel this article fully encapsulates the spirit of *FEBS Open Bio* – the article was originally submitted to *FEBS Letters* and sent for peer review. The manuscript was then offered the opportunity to transfer to *FEBS Open Bio*. The authors asked for advice from the academic community on Twitter and, after some discussion with the journal’s Managing Editor, were persuaded to complete the transfer. The manuscript was rapidly accepted based on the reviewer reports received from *FEBS Letters,* and we were delighted to see that the article has gained tremendous traction and is already well cited in the few months since its publication, confirming the importance of publishing these data. This case demonstrates the value of transferring reviewer reports between journals, which saves considerable time for reviewers, editors and authors.

## The year ahead

Last year saw many new developments for the journal, and we fully intend to continue and expand on these new initiatives in 2022. We are planning to publish several new ‘In the Limelight’ issues, including sections focussing on lysosomes, neurotransmitter release, virology, SARS‐CoV‐2 structure and RNA biology, so please look out for these later this year. In addition, we plan to continue publishing Insight articles and Interviews, with several in the pipeline already. On the more distant horizon, we are also planning to publish a special issue of articles authored by recipients of FEBS fellowships in 2023, to accompany the FEBS fellows meeting to be held in Lisbon in 2022. In addition, we are also developing a new article type, to be called ‘Research Protocols’. Such articles will provide comprehensive information on the materials and methods required to perform technically challenging experiments, with a level of detail greater than that provided in a standard article Methods section. In addition, these articles will provide tricks and tips for common troubleshooting issues, with the goal of helping to combat the reproducibility crisis by ensuring that full experimental details are available. We hope that this will be a valuable resource for the community, and we invite anyone interested in submitting such an article to contact the journal’s editorial office for further details.

We would like to thank everyone who submitted their work to the journal for consideration in 2021 and would like to invite all our readers to consider submitting to *FEBS Open Bio* in 2022. We have provided some reasons to submit to *FEBS Open Bio* below:

‐ Not for profit: Like the other FEBS Press journals (*The FEBS Journal, FEBS Letters* and *Molecular Oncology*), *FEBS Open Bio* is society‐owned and not for profit. Any profits made on the journal are reinvested into science by FEBS to support fellowships, advanced courses, the annual FEBS Congress, travel grants and many other activities. As such, by submitting your work to *FEBS Open Bio*, you are helping to support scientists across Europe and beyond.
‐ Science publishing, by scientists: All submissions are assessed by active researchers on our prestigious Editorial Board and are reviewed by members of our Editorial Advisory Board, volunteer reviewer pool or external reviewers, all of whom are experts in their respective fields.
‐ Fully open access: As a fully open access journal, all articles published in the journal are immediately free to read by everyone with an internet connection.
‐ Reasonably priced: We work hard to keep the article processing charge for authors as low as possible.
‐ Committed to sound science: We are committed to publishing sound science that makes a meaningful contribution to the field (that is, it should be a complete piece of work), and leave assessments of ‘impact’ to the community after publication.
‐ Speed: We are committed to ensuring that your manuscript is reviewed as quickly as possible, without sacrificing rigour in the peer‐review process. At the time of writing, the median time to first decision for submissions sent for peer review in 2021 was 28 days.
‐ Ease of transfers: Authors of manuscripts rejected by one of other three FEBS Press journals may be given the opportunity to easily transfer their work to *FEBS Open Bio*. Authors of such transferred manuscripts receive a discount on the article processing charge if their work is accepted.
‐ Figure screening service: All figures of accepted manuscripts are carefully screened by our image integrity analyst prior to publication to identify any errors (e.g. duplication of images).

Finally, we would like to thank all of our authors, colleagues, editors, readers and reviewers for your ongoing support and wish you all a productive and positive New Year.
